# Promoting Mental Health and Well-Being among Adolescent Young Carers in Europe: A Randomized Controlled Trial Protocol

**DOI:** 10.3390/ijerph18042045

**Published:** 2021-02-19

**Authors:** Giulia Casu, Valentina Hlebec, Licia Boccaletti, Irena Bolko, Alessandra Manattini, Elizabeth Hanson

**Affiliations:** 1 Department of Psychology, University of Bologna, 40127 Bologna, Italy; 2 Faculty of Social Sciences, University of Ljubljana, 1000 Ljubljana, Slovenia; irena.bolko@fdv.uni-lj.si; 3 Faculty of Health Sciences, University of Ljubljana, 1000 Ljubljana, Slovenia; 4 Anziani e Non Solo Società Cooperativa Sociale, 41012 Carpi, Italy; progetti@anzianienonsolo.it (L.B.); a.manattini@anzianienonsolo.it (A.M.); 5 Department of Health and Caring Sciences, Linnaeus University, SE-39182 Kalmar, Sweden; elizabeth.hanson@lnu.se

**Keywords:** adolescent young carers, primary prevention intervention, mental health, well-being, resilience, psychoeducational model, acceptance and commitment therapy, randomized controlled trial, study protocol, cross-national study

## Abstract

It is estimated that 4–8% of youth in Europe carry out substantial care for a family member or significant other. To prevent adverse psychosocial outcomes in young carers (YCs), primary prevention resilience building interventions have been recommended. We describe the study protocol of an international randomized controlled trial (RCT) of an innovative group intervention designed to promote the mental health and well-being of adolescent YCs (AYCs) aged 15–17. The RCT will be conducted in six European countries in the context of the Horizon 2020 European funded research and innovation project “Psychosocial support for promoting mental health and well-being among adolescent young caregivers in Europe” (“ME-WE”). The ME-WE intervention is based on Hayes and Ciarrochi’s psychoeducational model for adolescents and will consist of seven 2-h sessions in a group format, aimed to help AYCs build psychological flexibility and live according to their values. The control group will be a waitlist. Primary and secondary outcomes and control variables will be measured at baseline (T0), post-intervention (T1) and 3 months follow-up (T2). The COVID-19 pandemic has made amendments necessary to the original study protocol methodology, which we describe in detail. This study will contribute to building an evidence-based manualized program that educators and health and social care professionals can use to support AYCs in their transition to adulthood. From a research perspective, the outcomes of this study will contribute to evidence-based practices in primary prevention of psychosocial difficulties in AYCs and will gather novel knowledge on the effectiveness of Hayes and Ciarrochi’s model for use with middle adolescents with caring responsibilities. The trial has been preregistered (registration number: NCT04114864).

## 1. Introduction

Young carers (YCs) are people under 18 who take on significant or substantial caring tasks (e.g., household management, physical and emotional support and intimate personal care) often on a regular basis and assume a level of responsibility that would usually be associated with an adult [1]. They look after family member(s) (e.g., parents, siblings and grandparents) or a significant other such as a friend/schoolmate or neighbor. The person(s) they care for may suffer from a disability (e.g., physical disabilities caused by injury or illness), chronic physical (e.g., diabetes, asthma and cancer) and/or mental (e.g., depression, anxiety and schizophrenia) illness or substance use issue, neurodevelopmental (e.g., autism and intellectual disability) or neurological (e.g., dementia, stroke and epilepsy) disorders and/or frailty due to ageing, who require care, support and/or supervision [1,2]. It is estimated that in Europe, around 4–8% of children and young people aged between 10 and 24 years carry out substantial care for a family member or significant other [3].

Taking on care responsibilities early in life may have considerable negative consequences for young people’s mental and physical health and psychosocial development [4,5,6,7,8,9]. Caring has especially been linked to stress, anxiety and depression symptoms when occurring in the adolescent phase [10,11], which is remarkable given evidence that most mental health problems that start during adolescence subsequently persist into adulthood [12]. Risk factors that have been reported to affect physical and mental health outcomes of adolescent YCs (AYCs) include being female or non-binary and having a migration background [8,13], living with care recipients [8], the extent (i.e., greater amount of time) [13,14,15] and nature (i.e., personal and emotional care, financial and practical management) [7,14] of caring activities and food insecurity [16,17]. AYCs are also likely to face difficulties in education that negatively impact future employability, career aspirations and socioeconomic status [5,18,19,20]. Among risk factors for educational outcomes, more hours of care provided and caring for someone with a mental illness or using alcohol/drugs have been associated with lower emotional engagement (i.e., positive attitudes to teachers, classmates, schoolwork and the school environment) and greater problems with school in terms of both attendance and performance [21,22,23]. Furthermore, AYCs often report stigma leading to social isolation, bullying and victimization, which constitutes a risk factor for school absenteeism [24,25]. Altogether, caring responsibilities in the adolescent phase may have a serious impact on psychosocial adjustment and represent a challenge to life planning in relation to education, career and personal life [9,26]. However, self-efficacy beliefs, perceived social support, opportunities for leisure/recreation and meaningful social interactions and emotional engagement with school may act as protective factors, as they have been linked to better adjustment to the caring role, physical and psychosocial well-being and educational achievement [7,14,27,28]. Furthermore, resilience and a positive sense of self-identity have been suggested to promote coping with caring circumstances [29,30]. Among lifestyle factors, fruit and vegetable intake and physical activity have been associated with better mental health outcomes among adolescents and YCs [31,32].

Despite the potentially detrimental effects of caring on adolescents, psychosocial interventions to support AYCs worldwide are generally quite limited [33]. These include information and counseling-based services, support groups, socialization and respite care and skill-building programs [33,34,35,36,37,38,39,40]. In order to prevent an entrenched level of caring that can result in significant and long-term effects on YCs’ well-being and hinder their transitions to adulthood, it has been suggested that a primary prevention model should be adopted [18,38,39,40]. Specifically, it has been pointed that building the resilience of AYCs is especially important to avoid adverse mental health, social and educational outcomes [27,41]. Resilience, defined as the ability to recover from and adjust easily to adversity or unexpected changes, plays a fundamental protective role during the transitional phase of adolescence [42].

Resilience-building interventions for young people have been shown to promote social and emotional development and reduce psychological distress, with sustained effects [43,44]. However, to our knowledge, no resilience-based intervention for AYCs has yet been tested in Europe. In Australia, a resilience-based program for children (aged 12–18) of parents with a mental illness was evaluated [41]. However, allocation of the 44 participating AYCs to intervention or control groups was not randomized. More recently, a resilience-building program was tested among twelve Australian AYCs aged 12–14 [33]. Besides the small sample size, objective outcome measures, baseline or long-term follow-up assessment were not applied. These studies, while showing promising results, call for rigorous efficacy studies in Europe that involve larger samples, collect both quantitative and qualitative data, and consider both internal (e.g., mental health and well-being) and external (e.g., social support and school performance and attendance) outcomes [6,33,41].

Based on the above, the research project “Psychosocial support for promoting mental health and well-being among adolescent young caregivers in Europe” (ME-WE), funded by the European Union (Horizon 2020; 2018–2021), aims to develop an innovative framework of primary prevention interventions for promoting the mental health and well-being of AYCs aged 15–17, to be tested in six European countries (Italy, Slovenia, Sweden, Switzerland, the Netherlands and United Kingdom). Definitions of mental health are many and vary [45]. In the context of the ME-WE project, mental health has been conceptualized as a dynamic state of internal equilibrium, which enables individuals to utilize adaptively basic cognitive and emotional skills (such as recognizing, expressing and regulating one’s emotions), be flexible and cope with adverse life events and changes, function in social roles and participate in meaningful social interactions and maintain a harmonious relationship between body and mind [46]. An age range of between 15 and 17 years was selected as middle adolescence is often recognized as a critical period of transition into adulthood, in which adolescents deal with the creation and management of their identity and sense of self [47]. This time of intrapsychic changes has been associated with an increased susceptibility to the development of various mental health problems [48], calling for special attention to this age group.

The ME-WE primary prevention intervention program for AYCs has been developed based on the theoretical framework of the DNA-V model (discoverer, noticer, advisor and values) by Hayes and Ciarrochi [49]. The DNA-V model is an adaptation of the acceptance and commitment therapy (ACT) evidence-based approach [50], which is suitable for working with adolescents to promote their mental health and well-being. This psychoeducational group intervention is used in educational and clinical settings to help adolescents manage difficult emotions, find solutions to everyday problems, connect with their values, achieve mindfulness and vitality and develop positive relationships with friends and family. It focuses on developing a strong sense of self and giving adolescents the confidence that they need to make the transition into adulthood [49]. The model describes three functional classes of behavior, which are referred to using the metaphorical terms of discoverer, noticer and advisor. At the center of the model there are the values that guide behaviors. The discoverer involves behaviors related to exploring and testing the world through trial and error learning. The noticer is a powerful process that allows us to connect with our inner experience and the physical signals coming from the external environment. The advisor metaphorically represents our own “inner voice”, which is used to make sense of the past, form beliefs, evaluate ourselves and others without the need for trial and error. The discoverer, noticer and advisor provide the means to engage in values-based action. Values are conceptualized as a compass that guides individuals through the difficult moments of life and toward the things that are important to them. The ultimate goal of the DNA-V model is to build psychological flexibility, i.e., “the ability to utilize DNA skills in a way that promotes growth and builds vitality and valued action” [49] (p. 10), which is critical to resilience promotion [51,52]. Specifically, DNA-V promotes resilience by reinforcing adolescents’ ability to shift from the advisor’s worry and rumination about negative events into noticing and connecting with values in the present moment. DNA-V also helps young people to accept negative emotions as they are and without judgment rather than fighting against and amplifying them. Finally, DNA-V encourages youth to shift into the discoverer’s space to find new ways to move beyond adversity [49].

The DNA-V model was deemed to be especially coherent with the objectives of the ME-WE project, i.e., to promote the mental health and well-being of AYCs who are in the critical period of transition into adulthood. Indeed, the educational journey involved in the DNA-V model can assist AYCs in recognizing, accepting and sharing the emotions aroused by their caring experiences, which are often silenced by these youth [53]. Additionally, the DNA-V model has the potential to help AYCs explore and expand their behavioral repertoires to develop new and more effective ways of being in their caring role and in social relationships, to develop a flexible self-view and explore identities and future opportunities [26].

Meta-analytic and systematic review findings indicate that the ACT model, on which the DNA-V is grounded, is effective in the treatment of children and young people across a wide range of psychological and adjustment problems [54,55,56,57]. The DNA-V protocol has been piloted in cohort studies with clinical and non-clinical adolescents and showed promising results in terms of improved well-being and psychological flexibility [58,59,60]. However, its effectiveness has not yet been rigorously tested by means of a randomized controlled trial (RCT). In view of this research gap and the objectives of the ME-WE European project, the aim of this study was to design an international RCT to evaluate the effectiveness of the ME-WE intervention, which is an adaptation of the DNA-V model that accounts for the specificity of the target population of AYCs aged 15–17. Therefore, our primary research question was: Does the ME-WE intervention promote favorable changes in AYCs’ mental health and well-being outcomes compared to a control group? As a secondary research question, we aim to establish whether the ME-WE intervention leads to greater improvements in educational or vocational outcomes than a control group. To answer primary and secondary research questions, a number of primary and secondary outcomes, respectively, will be measured and compared between the study arms across the duration of the study (i.e., from baseline through post-intervention and 3 months follow-up). Primary outcomes will be those directly targeted by the ME-WE intervention, including psychological flexibility, mindfulness, resilience, mental and physical health, impact of caring and social support. Secondary outcomes will include self-reported functioning (i.e., experience, performance and attendance) at school/training/work, which are not directly targeted by the ME-WE intervention.

In the following sections we will first describe the original study protocol and then subsequent amendments to the research methods that were made necessary due to the COVID-19 pandemic.

## 2. Original Study Protocol Methods

### 2.1. Study Design

**Figure 1:**
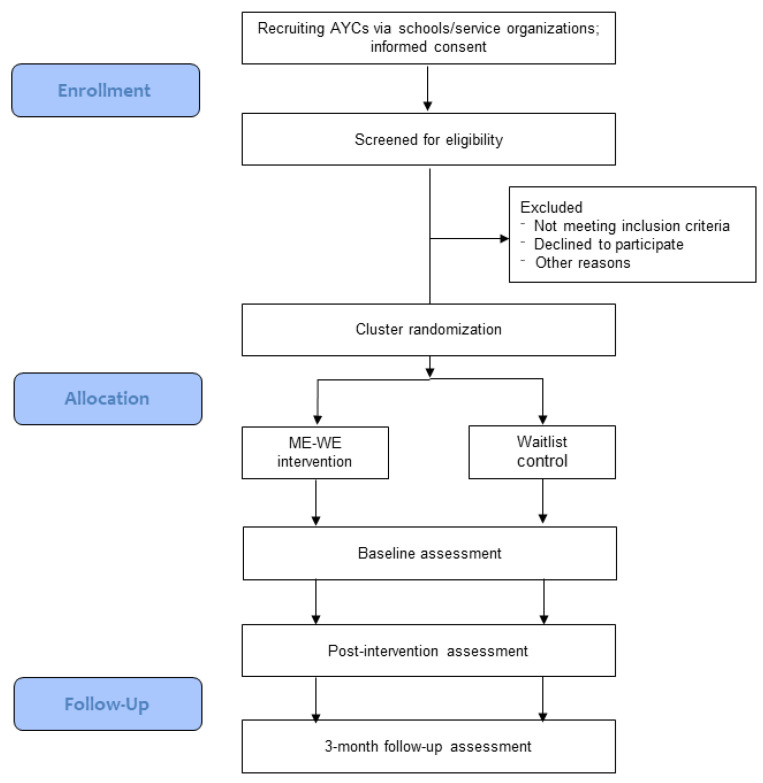
Consolidated standard of reporting trials (CONSORT) flow diagram for the original study protocol.

This study is a two-armed, parallel group cluster RCT that will be executed in six European countries (Italy, Slovenia, Sweden, Switzerland, the Netherlands and United Kingdom). Cluster randomization is used to minimize contamination between intervention and control arms [61]. Indeed, if randomization were performed at the individual participant level, there would be a risk for exchange of information between AYCs within the same cluster. Clusters will consist of AYCs attending the same school (in Slovenia, Sweden and Switzerland) or living in the same geographical area (i.e., neighborhood or post-code area) (in Italy, the Netherlands and the United Kingdom). Clusters will be randomly assigned to either the ME-WE intervention or the waitlist control (1:1 allocation ratio), so that all eligible participants from the same school or geographical area are in the same study arm. Randomization will be conducted by a research member who is neither involved in the recruitment nor in the intervention delivery, using an online number generator. To achieve a certain degree of blinding, both schools and community-based service organizations (for geographical area clusters) and participating AYCs will be informed that the study aims to investigate the effects of different support strategies on AYCs’ well-being. They will not be told which condition they have been allocated to, nor will they be offered detailed information about the respective other trial arm during the study. Outcomes will be measured at the individual level. Both the ME-WE intervention and the waitlist control group will be assessed at baseline (T0), immediately post-intervention for the ME-WE intervention group or after 7 weeks for the waitlist control group (T1), and at a 3 months follow-up (T2). Participant-reported outcome measures will be used. Trial data analysts will be blinded to group allocation. After the 3 months follow-up the waitlist control group will receive the same program as the intervention group. The consolidated standard of reporting trials (CONSORT) 2010 statement extended to cluster trials is followed in this study [62]. The research flow diagram is presented in Figure 1. This protocol is based on the SPIRIT (standard protocol items: recommendations for interventional trials) guidelines [63] and has been registered at clinicaltrials.gov (accessed on 22 September 2019, trial registration number: NCT04114864).

### 2.2. Participants

#### 2.2.1. Recruitment

All countries will employ cluster-targeted recruitment methods. Recruitment of AYCs will be performed in either schools only (in Slovenia, Sweden and Switzerland) or geographical areas by also partnering with community-based service organizations (e.g., community health and social service agencies and organizations and/or youth welfare agencies, carer-related or disease-specific non-governmental organizations and young carers charities) in addition to schools (in Italy, the Netherlands and the United Kingdom). Multiple recruitment methods will be used, including presentations of the research project in schools and youth centers, promotional materials such as leaflets and posters, social-networks, media and press releases and healthcare professionals’, social workers’ and teachers’ referrals.

A brief face-to-face or telephone screening interview will be conducted by the research team members to assess the eligibility of potential participants in each country. All participants will be provided with a plain language statement describing the general purpose of the study and required to provide written parental (or guardian) and self-consent to be involved in the study. Where only self-consent of participants is required according to national legislation, then participants’ legal representatives will be provided with information about the study and the young person’s participation. Participants will be informed of the voluntary nature of their participation and about their right to withdraw from the study at any time without having to provide a reason and without any adverse consequences.

#### 2.2.2. Inclusion and Exclusion Criteria

To be eligible for the present study, the following inclusion criteria have been established: (1) being between 15 and 17 years of age; (2) taking on caring tasks for family member(s) (e.g., parents, siblings and grandparents) or significant other (e.g., friend, schoolmate or neighbor) with a disability, chronic physical and/or mental health illness or substance use issue, neurodevelopmental or neurological disorders and/or problems related to old age [1,2]. Exclusion criteria will be: (1) concurrently participating in psychotherapies or mindfulness-based interventions/programs; (2) having started a new psychotropic medication within the past 30 days or planning on starting or changing psychotropic medication during the course of the study and (3) limited knowledge of the local language, with the exception of Sweden. The inclusion and exclusion criteria will be assessed at the screening interview conducted by the research team members.

### 2.3. Intervention

The ME-WE primary prevention intervention was developed by adjusting the existing DNA-V protocol [49] to the specific needs and experiences of AYCs aged 15–17. It was codesigned with YCs, former YCs and different types of professionals (e.g., psychologists, teachers, youth workers, health and social care professionals) in the framework of the blended learning networks (BLNs) performed at earlier stages of the ME-WE European project. BLNs are heterogeneous communities of practice that enable the voices of users and multistakeholders to be heard and that lead to shared learning [64]. In the ME-WE project, each country set up a BLN, which included between 8 and 14 participants (YCs, former YCs, professionals and representatives from non-governmental organizations) with the goal of contributing to the project implementation by providing their expert knowledge.

Participants of clusters allocated to the ME-WE intervention group will attend seven weekly 2-h group sessions, with a follow-up meeting after 3 months from the end of the intervention. Groups will be comprised of 5–10 AYCs. All sessions will have a similar structure (objectives, icebreaker, central activity/ies and final activity). After a first introductory session regarding the DNA-V model, sessions 2, 3 and 4 will introduce the advisor, noticer and discoverer metaphors, respectively. Session 5 will deal with values and values-oriented action and session 6 will attain psychological flexibility and self-compassion (i.e., the ability to approach personal suffering and failures with openness and awareness and showing self-kindness) [65]. The closing session 7 will focus on building strong social networks. At the end of sessions 2, 4, 5 and 6, participants will be provided with exercises to do at home, between one meeting and the next one, to practice the skills acquired during the session in everyday life. In the follow-up session, participants will reinforce the skills learned and discuss their experiences with the intervention. Table A1 provides a session-by-session outline of the ME-WE intervention contents.

Throughout the intervention, participants will learn to develop their noticer skills and thus to recognize and accept their internal experiences (thoughts, emotions, sensations and memories), including those related to caring, rather than reacting against or silencing them. They will become familiar with their advisor and become aware of the natural tendency of our mind to judge, evaluate and label, and will learn when to listen to and when to ignore it. They will develop their discoverer skills to experience new or alternative behaviors and build strengths. During the intervention, AYCs will also train some higher-order skills that emerge from DNA-V concerning their self-view and social view. They will learn to see themselves across different contexts beyond the caring one, to undermine unhelpful self-concepts and to develop self-awareness, self-knowledge and useful self-concepts. They will acquire perspective-taking skills beyond the self, to relationships and social groups, which will assist them in strengthening close relationships and building supportive social networks. They will also learn breathing and mindfulness exercises. The skills developed throughout the ME-WE intervention will ultimately help AYCs to be flexible in facing life events and live according to their values.

Two different methods will be followed in the delivery of the ME-WE intervention: a fully face-to-face approach (adopted by Italy, Slovenia and United Kingdom), and a blended approach that combines face-to-face and online sessions delivered via a dedicated ME-WE mobile app (adopted by Sweden, Switzerland and the Netherlands), which was codesigned with YCs at earlier stages of the ME-WE European project. Recent meta-analytic evidence supports the equivalence of face-to-face and web-mediated psychological interventions [66,67]. Both delivery approaches will address the same psychological processes and will use similar exercises and activities. The rationale for adopting two distinct approaches lies in different country characteristics with respect to the use of information and communication technologies. Indeed, recent data indicate that Sweden, Switzerland and the Netherlands rank among the highest in digital skills, with 72–79% of citizens having basic or above basic overall digital skills, while Italy and Slovenia rank among the lowest, with 42–55% [68]. Although United Kingdom ranks above average digital skills (74%), they opted for a face-to-face approach due to their prior experiences of running face-to-face groups in the context of YCs’ projects [69]. In the blended approach, sessions 1, 3, 7 and the 3 months follow-up meeting will be performed face-to-face, while sessions 2, 4, 5 and 6 will be performed online using the ME-WE app and the ZOOM platform. In the online sessions, the theoretical DNA-V inputs will be provided through short videos. The activities and exercises have been slightly modified to be easily performed online and remotely by participants, but facilitators will use the same instructions as in the fully face-to-face approach.

In both delivery approaches, two facilitators will conduct each session. Facilitators will be psychologists, youth workers, teachers, school nurses and carer support workers, all with solid experiences in working with groups and with young people. All facilitators will be trained in the ME-WE intervention before conducting the sessions, including information on the theoretical background of the DNA-V model and training sessions on the ME-WE intervention exercises that use a role-play technique. Facilitators will also be provided with both the DNA-V protocol manual and/or training course [70] and the ME-WE intervention manual (translated from English into local languages by local team members who are fluent in English). Throughout the study duration, they will be provided with ongoing supervision.

#### Intervention Fidelity

Fidelity measures will be employed to ensure consistent delivery of the ME-WE intervention across facilitators and locations. First, it is a requirement that all facilitators have experience working with groups and young people. Second, role-playing exercises will be applied in the training of facilitators to have them practice and increase their confidence in the implementation of the intervention. Third, a standardized, detailed intervention manual will be available to facilitators. Fourth, following the delivery of each session, cofacilitators will complete a semistructured questionnaire to evaluate adherence to the intervention manual, skills in creating a positive group environment and participants’ engagement. Finally, facilitators will receive monthly supervision from a mental health professional trained in the DNA-V to ensure intervention fidelity and to offer supplemental supervisory support as needed.

### 2.4. Control

Participants of clusters allocated to the waitlist control group will not receive an intervention. They will perform ice-breaker and team building activities during 3 face-to-face meetings organized to correspond with the three assessment points with the aim of collecting outcome measures data. They will be offered to receive the ME-WE intervention after the experimental group has completed all the assessments (i.e., after the 3 months follow-up).

### 2.5. Outcome Measures

Demographic (gender, age, country of birth, nationality, migration background, place of living, living conditions and family composition) and caregiving information (number and age of people cared for, type of relationship with the care recipient, nature of the illness or health condition of the care recipient and time since caregiving started) will be collected at baseline (T0). Primary and secondary outcomes and control variables will be collected at baseline (T0), post-intervention (T1) and 3 months post-intervention (T2). T0, T1 and T2 assessments in the ME-WE intervention group will be performed at the beginning of session 1, at the end of session 7 and at the beginning of session 8, respectively. Primary outcome variables will include psychological flexibility, mindfulness skills, resilience, subjective mental and physical health, general and caring-related quality of life, impact of caring and social support. Secondary outcome variables will be self-reported school/training/work experience, performance and attendance. The following variables will be used as control variables: caring activities, overall amount of caring, likes and dislikes of caring, formal support received and caring-related perceived social support. Both quantitative and qualitative data will be collected using a questionnaire available in both web-based and paper-and pencil versions. All selected standardized self-report measures were specifically developed and/or tested for use with adolescents. Measures not available in the local languages of participating countries have been translated after permission from the authors. The committee translation method was used [71]. Specifically, in non-English countries, two independent researchers fluent in English independently translated each measure from English into their local language, and then met to discuss eventual discrepancies and reach consensus on an integrated version. English questionnaires were used for participants in the United Kingdom. Ad-hoc developed items were formulated in English and then translated using the same procedure used for standardized tools. Participants in the intervention group will complete a post-intervention self-assessment tool at T1 and T2. Process evaluation outcomes will include feasibility, acceptability and fidelity indicators.

#### 2.5.1. Primary Outcomes

Psychological flexibility will be assessed using the avoidance and fusion questionnaire for youth (AFQ-Y) [72] (8 items, 5-point scale ranging from not at all true to very true). The AFQ-Y has been developed based on the ACT model and validated for use with youth into several languages, including Italian [73], Dutch [74] and Swedish [75,76]. All versions showed adequate reliability and concurrent validity with measures of internalizing and externalizing symptoms [72,73,74,75,76]. Mindfulness skills will be measured with the child and adolescent mindfulness measure (CAMM) [77] (10 items, 5-point scale ranging from never true to always true). The original CAMM has been validated in a large sample of children and adolescents, showing a one-factor structure with adequate internal consistency and associations in the expected directions with a number of criterion measures (e.g., quality of life and internalizing and externalizing problems) [77]. The validated Italian [78,79,80] and Dutch [81] versions used in this study demonstrated sound reliability and construct validity. Both the AFQ-Y and CAMM showed sensitivity to change when used with children and adolescents receiving ACT- or mindfulness-based interventions, e.g., [57,82,83,84,85]. To assess resilience, the brief resilience scale (BRS) [86] (6 items, 5-point scale ranging from strongly disagree to strongly agree) will be applied. The original BRS showed evidence of validity and reliability as a one-factor measure of the ability to recover from stress and has already been used in research with AYCs [87]. The Dutch [88] and German [89] adaptations used in this study demonstrated adequate reliability, meaningful associations with mental health symptoms and confirmed the BRS original structure. The Warwick Edinburgh mental well-being scale (WEMWBS) [90] (14 items, 5-point scale ranging from none of the time to all of the time) will be used for measuring subjective mental health. The original WEMWBS was developed in the United Kingdom and showed sound psychometric proprieties including internal consistency and evidence of validity based on content, internal structure and relations with other mental health and well-being scales [90]. This widely used measure has been tested for use with adolescents [91] and proved valid and reliable in its Italian [92], Dutch [93], Slovenian [94], Swedish (7-item WEMWBS) [95] and German [96] adaptations. Subjective physical health will be assessed with the health behavior in school-aged children symptom checklist (HBSC-SCL) [97] (8 items, 5-point scale ranging from about every day to rarely or never). The HBSC-SCL has been developed within the HBSC study, a World Health Organization collaborative cross-national study of adolescent health and well-being that includes fifty countries and regions across Europe and North America, including the six countries of the present study [98]. Validation studies on the HBSC-SCL indicate that it measures two dimensions of subjective health complaints (i.e., psychological and somatic) with adequate internal consistency and test–retest reliability, and content, convergent and discriminant validity [99,100]. For general quality of life, the KIDSCREEN-10 [101] (10 items, 5-point scale ranging from not at all/never to extremely/always) will be employed, with an additional item on subjective general health (5-point scale ranging from excellent to poor) and one multiple-choice ad-hoc question on currently experiencing health-related issues (i.e., mental health problems, physical disabilities, learning difficulties or other). The KIDSCREEN-10 was developed from the 27-item KIDSCREEN to obtain a Rasch-scaled single score of quality of life for children and adolescents. It was validated in a large sample of youth across thirteen European countries, including Italy, Netherlands, Sweden, Switzerland and United Kingdom, showing adequate reliability and construct and criterion validity [102]. The KIDSCREEN-10 has been adopted in the HBSC study and has been used in research on AYCs [13,103]. The translations used in this study were taken from [104]. Caring-related quality of life will be assessed using 3 dichotomous (yes/no) ad-hoc questions regarding thoughts about hurting oneself and others, and being bullied, teased or made fun of, and one multiple-choice ad-hoc question on experienced health-related issues (i.e., mental health problems, physical health problems or other) because of the caring role. Perceived cognitive and emotional impact of caring will be assessed using the positive and negative outcomes of caring (PANOC) [105] (20 items, 3-point scale ranging from never to a lot of the time). The PANOC was specifically developed for and validated in YCs in the United Kingdom. It consists of two 10-item subscales (positive and negative outcomes) with adequate internal consistency and expected associations with depressive symptoms [105]. A Swedish adaptation has been used in previous studies, showing adequate internal consistency [6]. Finally, perceived social support will be measured using an adapted version of the brief social support questionnaire (SSQ-6) [106] that includes 3 items asking to indicate the number of support sources and one item asking for global satisfaction with received social support (6-point scale ranging from very dissatisfied to very satisfied), and one multiple-choice ad-hoc question on individuals (i.e., school staff, close family members and close friends) AYCs can count on if in need of support or help. The original SSQ-6 validation study indicated a two-factor structure (availability of and satisfaction with social support) with adequate reliability and expected relations with personality dimensions [106]. The Dutch adaptation showed adequate reliability [107]. The SSQ-6 has been previously used with adolescent populations [108] and with AYCs showing good reliability and meaningful associations with indicators of well-being [27,109].

#### 2.5.2. Secondary Outcomes

Self-reported school, training or work experience, performance and attendance will be measured using 5 ad-hoc items asking for perceived caring-related difficulties in education/training/work (e.g., “I miss school/training/work”; “It is difficult for me to perform well at school/training/work”; “While at school/training/work, I worry about the person I care for”). Items are rated on a 5-point frequency scale (from never to always). Three ad-hoc items (5-point scale ranging from yes, absolutely to absolutely not) will assess likeliness to complete and continue education/training compared to before participation.

#### 2.5.3. Control Variables

Caring activities will be measured using the multidimensional assessment of caring activities [105] (18 items, 3-point scale ranging from never to a lot of the time), which asks for time spent on domestic tasks, household management, personal care, emotional care, sibling care and financial/practical care. To assess the overall amount of caring, 2 questions taken from the “Manual for Measures of Caring Activities and Outcomes for Children and Young People” [110] will be used that ask for the number of hours of caring per week for a typical day during the week and at the weekend. To assess likes and dislikes about caring, 4 open-ended questions based on the Joseph et al. manual [110] will be used that ask AYCs, which one of their caring jobs they like the most, dislike the most, that gratifies them the most or upsets them the most. Three multiple-choice ad-hoc questions will assess formal support or services that AYCs and someone in their family receive (i.e., care package, equipment, transportation assistance, benefits and allowances), and whether school staff, other family members and friends have been trusted or know about their caring situation.

#### 2.5.4. Post-Intervention Self-Assessment

At both T1 and T2, AYCs allocated to the ME-WE intervention group will be asked to complete an adaptation of the post intervention self-assessment by Joseph et al. [110]. This tool includes 7 dichotomous (Yes/No) items on the ME-WE intervention (e.g., “I enjoyed most of the activities”), 10 items (3-point scale ranging from more often than before the intervention to less often than before the intervention) about changes related to participation (e.g., “I feel able to choose the level of care I provide”) and 5 open-ended questions (see next section).

#### 2.5.5. Process Evaluation Outcomes

A number of process evaluation outcomes will be monitored, following Linnan and Steckler [111]. Indicators of feasibility will include the proportion of eligible AYCs who will participate in the trial, the proportion of AYCs in the intervention group who will attend ≥70% of intervention sessions, and the proportion of AYCs who will be lost to follow-up. These feasibility indicators will be assessed using attendance lists (for the intervention group) and completion of assessment questionnaires (for the control group). To assess intervention acceptability, 5 open-ended questions included in the post-intervention self-assessment [110] will ask AYCs for their perceptions about the support they received from the intervention, what changed for them and for their caring role because of participating in the intervention and what they did not like about the intervention. For fidelity, a semistructured questionnaire will be completed by cofacilitators following each intervention session to evaluate whether the ME-WE intervention was delivered as planned.

### 2.6. Piloting of the ME-WE Intervention and Assessment Questionnaires

Between March and May 2019, a small pilot study was carried out in Italy and the Netherlands with groups of 5 and 4 AYCs, respectively, to assess the suitability of the ME-WE intervention for our target group. Participants’ feedbacks were collected in group discussions with questions related to their satisfaction with intervention overall arrangement, timing and contents, and to individual experiences during session and home activities. Altogether, timing and contents were rated favorably, and intervention activities and exercises were well received among AYCs. Only minor adjustments were proposed, such as the need for introducing physical materials used (e.g., cards) and emphasizing that there are no correct or incorrect interpretations of the stimuli proposed in the session exercises, and more clearly explaining home activities and their voluntary nature. Proposed adjustments were subsequently implemented. In May 2019, the assessment questionnaire was piloted with two groups of AYCs from Italy (*n* = 4) and the United Kingdom (*n* = 5) to evaluate comprehensibility, acceptability and completion time. In general, the tool was deemed comprehensive and easy to understand and respond. Mean questionnaire completion time was 20 min. Altogether, the pilot studies provided useful information on practicality and suitability of the study materials.

### 2.7. Sample Size Calculation

For the primary outcome variables, a moderate effect size (*f* = 0.25) was pursued based on previous evidence in RCTs using ACT-based interventions [56,84,112]. A minimum group size of 24 AYCs was established based on power analyses with a power of 80% and an α-error probability of 5%. However, in the case of cluster RCTs, an effective sample size is in fact lower than that suggested by the actual number of participants due to observations on participants in the same cluster tending to be correlated [62]. Thus, initial sample size estimation (performed as if randomization occurred at the individual level) was corrected taking into account randomization occurring at the cluster level. For each country, an average cluster size was estimated using the formula for sample size determination in a finite population [113] and assuming the prevalence rate of AYCs in the population of adolescents aged 15–17 in that country. Based on available data, the estimated prevalence rate of YCs is about 3% in Italy [114], 7% in Sweden [115], about 8% in Switzerland [3], 6% in the Netherlands [116] and in the United Kingdom approximately 7% of all children are engaged in a high amount of caring and 3% in a very high amount [117], while no estimation is available yet for Slovenia. As Italy and Slovenia have both been classified as countries at the initial stage of awareness concerning AYCs [118], the Italian prevalence rate was also applied in the Slovenian sample size calculation. A design effect was then calculated using the average cluster size and an assumed intracluster correlation of 0.05. This design effect was multiplied by the initial sample size to obtain a sample size corrected for the cluster-based design, which was further increased by 20% to compensate for attrition. The minimum total sample sizes obtained from this procedure were as follows: 80 AYCs (40 AYCs in the intervention and 40 AYCs in the waitlist group) in Italy and Sweden, 76 AYCs (38 AYCs in the intervention and 38 AYCs in the waitlist group) in Slovenia, 102 AYCs (51 AYCs in the intervention and 51 AYCs in the waitlist group) in Switzerland, 112 AYCs in the Netherlands (56 AYCs in the intervention and 56 AYCs in the waitlist group) and 142 AYCs (71 AYCs in the intervention and 71 AYCs in the waitlist group) in the United Kingdom.

### 2.8. Statistical Analyses

Statistical analyses will be performed at both national and cross-national levels. Data will be analyzed using intention-to-treat principles [119] and adjusted for cluster effect [62]. Baseline characteristics will be preliminarily compared to check equivalence between study groups using analysis of variance (ANOVA) and *χ*^2^ tests. To answer primary and secondary research questions, differences in primary and secondary outcomes between the study arms from T0 through T1 and T2 will be tested using linear mixed effect models. These analyses will be based on individual participant-level data, allowing for clustering between AYCs within the same school or geographical area [62,120]. Models will include the fixed effects of study arm, country and delivery approach, time and their interaction effects. The cluster will be considered as a random effect. Models will be adjusted for characteristics that are unbalanced between study arms and significantly associated with the outcome measures. In case of violations of assumptions underlying planned analyses (e.g., equal variance and normal distribution of residuals) appropriate mathematical transformations of raw scores (e.g., logarithmic scale) or robust estimation methods will be applied [121]. Interpretation of results will be based on both statistical significance (*p* < 0.05, two-tailed) and measures of effect size [122]. We will also summarize post-intervention self-assessment and process evaluation measures. Analysis of qualitative data in national languages (for control variables likes and dislikes about caring and for intervention acceptability) will be performed using content analysis [123].

### 2.9. Ethics Approval

All participants will be involved in the study on a voluntary basis in accordance with the Declaration of Helsinki (1964; 2013) [124]. Informed consent will be sought from both AYCs and parents or legal guardians, in accordance with applicable national legislation and institutional guidance. Data will be processed in compliance with both national laws on data protection and the General Data Protection Regulation (GDPR) of the European Union (2016/679) [125] to guarantee the respondents’ anonymity and privacy. Formal ethics approval has been obtained from the Ethical Review boards of the University of Bologna (Italy), University of Ljubljana (Slovenia), University of Amsterdam, Faculty of Social Sciences (The Netherlands), University of Sussex (United Kingdom) and from the National Research Ethical Review Board (Sweden). According to the national human research act, the Ethics Committee of the Zürich Canton deemed formal ethical approval unnecessary.

## 3. COVID-19-Related Amendments to Study Protocol Methods

The COVID-19 pandemic has introduced important challenges to the continuation of this study. To avoid costly trial closures, deviations from the original study protocol were deemed unavoidable by the research project team and amendments were made to the originally planned methodology. To comply with the restrictions and precautionary measures introduced at national levels, the study has been virtualized on April 2020, including remote enrollment, screening, consent and data collection, and a fully online delivery of the ME-WE intervention and of training and supervision of facilitators [126,127]. COVID-19-related amendments to the study protocol methods are described in detail in the following subsections. If not specified below, the original study protocol as described in Section 2 will be followed.

### 3.1. Study Design Amendments

Sweden and Switzerland will turn to randomization of individual participants since their new recruitment method (see next section) does not pose a risk for spillover effects.

### 3.2. Participants

#### Recruitment Amendments

Due to COVID-related interruptions and slowing down of recruitment of trial participants, Sweden and Switzerland will launch national social media recruitment campaigns. In all six participating countries, recruitment and enrollment will be performed remotely, and screening interviews to assess eligibility of participants will be conducted exclusively by telephone or using video-conferencing applications (e.g., Microsoft Teams, ZOOM). Written parental (or guardian) and self-consent to be involved in the study will be collected by e-mail or recorded by Microsoft Teams (version 1.3) or ZOOM (version 5).

### 3.3. Intervention Amendments

In all six participating countries, new delivery methods will replace those originally planned for the ME-WE intervention. Specifically, fully face-to-face delivery (planned for Italy, Slovenia and United Kingdom) will be replaced by online sessions using video-conferencing instruments allowing for visual presentations of participants and session materials (e.g., Microsoft Teams and ZOOM). The blended delivery approach (planned for Sweden, Switzerland and the Netherlands) will be replaced solely by online meetings using the ME-WE mobile app and supported with the ZOOM video-conferencing system for all intervention sessions. In both delivery approaches, no changes will be introduced in the intervention contents. Some activities and exercises will be slightly adapted to fit the online delivery mode (Table S1).

Training and supervision of facilitators will be delivered fully online using video-conferencing platforms.

### 3.4. Outcome Measures Amendments

Assessments will be performed solely online, using the web-based version of the study questionnaires. Additional COVID-related measures will be included. Specifically, the perceived impact of the COVID-19 pandemic on everyday life and perceived mental health [128] will be assessed at all assessment points in both study groups. At T1 and T2, AYCs in the intervention group will be also asked for their experiences with participating in the ME-WE intervention during the pandemic and their evaluation of the online delivery mode.

#### 3.4.1. COVID-19-Related Control Variables

Three open-ended questions will ask AYCs for the impact of the COVID-19 pandemic on their lives and mental and physical health, and whether they or their families are receiving the support and services they need during the COVID-19 crisis. AYCs in the intervention group will receive a further open-ended question asking them how they experienced their participation in the ME-WE sessions and the exercises and home activities proposed in the ME-WE intervention during the COVID-19 pandemic.

#### 3.4.2. Evaluation of Online Delivery of the ME-WE Intervention

Satisfaction with the online delivery of the ME-WE intervention due to COVID-19 restrictions will be assessed. A Likert-type item (0 = totally dissatisfied to 10 = totally satisfied) will be administered, in addition to 3 multiple-choice items addressing satisfaction and dissatisfaction with specific features (e.g., user-friendliness and contents) and any problems encountered during the intervention delivery (e.g., network issues). Furthermore, participants will be asked how they felt about being part of a group (1 item, 5-point scale ranging from always to not at all). For groups that started face-to-face but then continued remotely due to COVID-19 outbreak amidst the intervention delivery, information on the perceived usefulness and enjoyability of online vs. face-to-face sessions will be also collected (2 items, 5-point scale ranging from always to not at all).

### 3.5. Sample Size Calculation Amendments

The minimum required sample size was revised for Sweden and Switzerland, since randomization in these countries will occur at the individual participant level. A minimum group size of 24 AYCs was established based on power analyses with a medium effect size, a power of 80% and an α-error probability of 5%. Compensating for attrition (20%), a minimum total sample size of 58 AYCs (29 AYCs in the intervention and 29 AYCs in the waitlist group) will be required in Sweden and Switzerland.

### 3.6. Statistical Analyses Amendments

For countries that turned to an individual-based RCT (i.e., Sweden and Switzerland), statistical analyses will be performed at the individual participant-level, with no cluster-based adjustment. Statistical analyses at the cross-national level will be performed separately for countries conducting cluster RCTs (i.e., Italy, Slovenia, the Netherlands and United Kingdom) and countries conducting individual-based RCTs (i.e., Sweden and Switzerland). For individual based RCTs, differences in primary and secondary outcomes between the study arms from T0 through T1 and T2 will be tested using mixed ANOVA. Models will include the effects of study arm, country, time and their interaction effects. In case of violations of assumptions underlying planned analyses, appropriate data transformations or robust estimation methods will be applied [121]. Analysis of qualitative data collected using newly added COVID-19-related open-ended questions in national languages would be performed using content analysis [123].

### 3.7. Ethics Approval of Amendments

The amendments introduced due to the COVID-19 pandemic have received formal ethical approval and/or detailed opinions (as appropriate according to national legislation) from the previously consulted Ethics Review boards in all six countries. The registered study protocol has been updated with protocol amendments at clinicaltrials.gov (accessed on 6 November 2020) following ethics approvals.

## 4. Trial Status

The recruitment of participants started in May 2019. Randomization began in September 2019, and the first baseline assessment was completed on 24 October 2019. Before COVID-19-related amendments (April 2020), a total of 98 AYCs was recruited. The trial is currently ongoing. Preliminary data available from Italy (*n* = 22, October 2019–March 2020) indicated feasibility of the ME-WE trial and acceptability of the ME-WE intervention. The proportion of eligible AYCs (screened between May and September 2019) who participated in the trial was 84%. Seventy-three percent of AYCs in the intervention group attended at least 70% of intervention sessions. Twenty-eight percent of all participants were lost to follow-up. Responses of AYCs in the intervention group to the post intervention self-assessment indicated that participants had a clearer vision of their thoughts, took better care of themselves and were generally satisfied with and enjoyed the intervention.

## 5. Discussion

AYCs are at risk of adverse mental, social and educational outcomes that may continue into adulthood implying reduced psychosocial adjustment [5,9,10,11,18,19,20,24,25]. A primary prevention model has been recommended to mitigate against negative outcomes and foster transitions to adulthood [18,38,39,40]. To the best of our knowledge, this study will be the first RCT evaluating the efficacy of a primary prevention, group-based intervention for middle adolescents who care for a family member or significant other in Europe. Noteworthy, participatory codesign, user-centered principles were followed in the development of the ME-WE intervention. In all participating countries, YCs, former YCs and professionals working in the field of youth and/or caring participated in BLNs aimed to collect expert knowledge for the designing of a support program tailored to AYCs’ needs and preferences, with expected favorable effects on participation, adherence and the likelihood of the program success. The components of DNA-V included in the ME-WE intervention promote skills to manage difficult emotions and thoughts, identify values and derive values-oriented goals, develop strengths, build positive social networks and achieve psychological flexibility and mindfulness [49]. The ME-WE intervention has the potential to prevent maladjustment and promote well-being and healthy development in AYCs by building resilience through the skills learnt to be flexible in facing life events, including caring-related ones, and live according to personal values. We anticipate improvements in protective factors targeted by the ME-WE intervention. Specifically, we expect that, compared to the waitlist control group, ME-WE intervention participants will report greater improvements in psychological flexibility, mindfulness, resilience, subjective health and quality of life and in perceived emotional impact of caring and social support, and that these effects will be maintained at the 3 months follow-up. We will also explore potential changes in self-reported school, training or work experience, performance and attendance, which are considered as secondary outcomes because the ME-WE intervention does not address these variables directly. This international RCT has received formal ethical approval and is now being implemented. On completion, results will be reported as outlined in the CONSORT statement [129] and will be published in subsequent peer-reviewed scientific publications, presented at national and international conferences and widely disseminated to professionals, decision makers, policy makers and civil societies at regional, national and international levels. In this article we also detailed the statistical analysis plan to ensure analytical transparency and avoid data-driven analyses [130,131].

If the ME-WE intervention proves its superiority, an evidence-based primary prevention group intervention to support AYCs’ well-being during their transition into adulthood would be available in both face-to-face and online, app-mediated versions. Such an intervention could be implemented in educational and clinical settings, and educators together with health and social care professionals will have a new avenue for referral of AYCs available to them. The availability of an app-based version has the advantage of reducing stigma and enhancing anonymity that may prevent adolescents from accessing psychosocial support services and programs [132]. As well, it provides greater flexibility as it is not bound to a physical location. Further, group-based interventions can be more cost-effective than one-to-one interventions, and have the valuable potential to reduce isolation, promote peer-to-peer interactions and create bonds among AYCs, which has been reported as an unmet need of these youth [133]. From a research perspective, the outcomes of this study will contribute to evidence-based practices in primary prevention of psychosocial difficulties in AYCs. Additionally, if the ME-WE intervention is found to have an effect, this study will gather novel knowledge on the effectiveness of the DNA-V model [49] for promoting the mental health and well-being of middle adolescents who care for a family member or significant other.

Our study design has some limitations that must be acknowledged. First, there is the lack of comparison with an evidence-based control intervention. However, an intervention for YCs in the middle adolescent phase has not previously been tested in Europe. Second, a cross-over design would ensure that participants in the waitlist group receive the intervention, besides providing strengthened statistical power. However, our choice of using a parallel group design was based on practical and methodological considerations. A cross-over design requires that participants receive one treatment first and then crossover to the other after a washout period; thus, it takes at least twice as long as an independent parallel group design, depending on the duration of the washout period [134]. The ME-WE project consortium deemed that a longer intervention period extended by the washout period was not feasible within the timeframe of the ME-WE funded project period. Furthermore, use of cross-over designs in trials testing non-pharmacological treatments is uncommon because the washout period of such interventions cannot be accurately evaluated, which poses risks for bias in outcome assessment [135]. Third, blinding of facilitators providing the ME-WE intervention is not possible, yet this is a general problem in psychosocial intervention trials [136]. Fourth, the study design does not allow one to identify components of the intervention that are most important in achieving change due to their synergetic and cumulative nature. Accordingly, the aim of this study was to determine whether the whole ME-WE intervention was more effective than a waitlist control in improving the outcome variables. Fifth, we would rely only on self-reported data. Additionally, we did not assess factors that might influence YCs’ mental health, such as food insecurity and dietary and physical activity habits [16,17,31,32]. However, we included overall amount of caring as a control variable, since there is evidence that YCs may lack sufficient time to engage in healthy lifestyle behaviors [137,138]. Noteworthy, we tried to keep the assessment questionnaires to a reasonable length to limit respondents’ burden. Inclusion of such protective and risk factors should be considered in future evaluations of the ME-WE intervention. Finally, only AYCs aged 15–17 were included in this study, thus the results will not be automatically generalizable to other age groups.

Despite such limitations, important strengths of this study include its international nature, and the development of the ME-WE intervention based on evidence-based techniques and components such as those in the DNA-V and ACT models [54,55,56,57]. Bias is minimized by the use of a manualized intervention, different facilitators, multiple assessment points and use of valid measures for primary outcomes. Combination of both quantitative and qualitative measures also constitutes a major strength, as it will allow for a more comprehensive interpretation of findings [139].

The COVID-19 pandemic has posed considerable challenges to this study. We followed recently proposed recommendations to reduce costly errors and to preserve ongoing trials [126]. Significant efforts have been made by the research team to adapt the study protocol and ensure study continuation while complying with the public health preventive measures. Amendments to the study protocol have been registered that include remote enrollment, screening and consent procedures, training and supervision of facilitators, intervention provision and data collection, and the inclusion of COVID-19-related variables in the assessment questionnaires. In this regard, we included questions on the perceived impact of the COVID-19 pandemic on AYCs’ lives and perceived mental health. Indeed, there is evidence that the COVID-19 pandemic had negative repercussions on the mental health and well-being of family carers of all ages [140], and worsening of mental health and increased social isolation were especially observed in AYCs [141,142]. Due to COVID-19-related school closures, AYCs were deprived of a form of respite where they can focus on other aspects of their lives [27], and reported an overall increase in time spent caring [141,142].

Despite COVID-19-related adjustments, we recognize that there may be potential difficulties in reaching the target sample size. Anticipated difficulties include recruitment problems due to stringent inclusion criteria, especially in light of the specific age-range of eligible AYCs. As well, a general lack of awareness and knowledge around the issue of AYCs in most participating countries [118], which might constitute a barrier in identifying YCs. Furthermore, given their caring responsibilities, it may be a challenge for AYCs to engage in a 7-week program, and more so in the midst of the COVID-19 pandemic [141,142]. To avert these anticipated concerns, a number of mitigation strategies will be adopted. Measures to facilitate recruitment include contacting additional potential referrers, arranging individual intake meetings with AYCs, and offering incentives (gift cards) for participation. Measures to promote retention of participants will include provision of as much logistic support as possible, regular communication and reminders and encouragement of socialization between AYCs in the intervention group.

## 6. Conclusions

AYCs tend to face significant adversity in their personal and social lives related to their caring role [26]. This is the first international RCT that aims to investigate the efficacy of a primary prevention intervention, based on the DNA-V model [49], in promoting the mental health and well-being of AYCs aged 15–17 in Europe. If the ME-WE intervention is successful, it will improve the psychosocial adjustment of AYCs by providing them with skills that can help to increase their resilience at times of adversity, which might assist them in their transition into adulthood. An evidence-based, manualized program for middle adolescents who carry out care for a family member or significant other is expected to fill a gap in the support offered to these youth at the European level. It may also lead, in the long run, to considerable savings in health and social care services and societal costs.

## Data Availability

The data presented in this study protocol are available from the corresponding authors on reasonable request. The data are not publicly available due their sensitive nature.

## Supplementary Materials

The following are available online at https://www.mdpi.com/1660-4601/18/4/2045/s1, Table S1: Session-by-session outline of the ME-WE intervention program for COVID-19-related online delivery.

## Appendix A

**Table A1:** Session-by-Session Outline of the ME-WE Intervention Program.

Session	Content
Session 1. Getting to know each other and intervention presentation.	Welcome, introduction of facilitators and participants; establishing program engagement and group rules. Introduction to DNA-V model and overview of the intervention; collecting participants’ expectations. Exercises: “DNA-V model description” ^1^, “Dropping the anchor”.
Session 2. The Advisor: dealing with annoying thoughts.	Introduction to the concept of annoying thoughts and the futility of trying to control them; learning to have a different, healthier relationship with these thoughts: letting them go in and out without being trapped in them; learning not to identify oneself with Advisor’s statements; introduction to the concept of mindfulness and first practice. Introduction to the online environment of the ME-WE app. ^2^ Exercises: “Draw and give a name to your Advisor” ^3^, “Give the Advisor a microphone” ^1^, “Normalize the Advisor—The GPS metaphor”^1^, “Going to extremes” ^1^, “Balloon breathing” ^4^. Home activity: “Watching for the Advisor” ^1^.
Session 3. The Noticer: being in connection with our feelings.	Identifying and getting in touch with our emotions, the body and the physical signals that come from the world around us, and understanding the importance of connecting with the present moment; developing understanding about the futility of trying to control unwanted feelings; learning to acknowledge feelings and allow them to be there; introduction to and practice on the AND acronym (Aware, Name, Describe). Exercises: “Rediscovering the wisdom of our feelings”, “Feeling with the body—Practicing AND” ^1^, “Seaweed goes with the ocean” ^1^.
Session 4. The Discoverer: growing and thriving.	Identifying challenging situations, encouraging the enlargement of the behavioral repertoire to make life bigger, richer, and more vital, or to refocus energy on better listening to what is important and taking committed action towards values; identifying personal strengths. Exercises: “Tracking workability of old behaviors” ^1^, “Strength spotting card sort” ^1^, “One moment meditation” ^4^. Home activity: “Using the Discoverer to discover values” ^1^.
Session 5. Values: connecting to meaning and vitality.	Introduction to values, and recognition of the important role they play on one’s life; learning to be free to listen to values and decide on actions; becoming aware of what is important in life, identifying values and goals, and committing to take action to live in line with them by developing an action plan. Exercises: “What values are” ^4^, “Values card sort” ^1^, “My valued journey” ^1^, “Mindfulness and music” ^4^. Home activity: “My valued journey”^1^.
Session 6. Developing a flexible self-view and self-compassion.	Developing a flexible self-view by experiencing oneself as a changing, evolving human, and weakening dysfunctional self-concepts; practicing loving-kindness toward oneself and self-compassion by learning to forgive oneself and working on guilt and self-criticism. Exercises: “Strengthening my self-view” ^1^, “Becoming a friend to yourself” ^1^, “Compassionate letter” ^4^, “3 min breathing space”^3^, “A gentle action”. Home activity: “A gentle action”.
Session 7. Building strong social networks.	Identifying what is important in a relationship and especially in a caring one; building strong social networks by becoming aware of people who can support and can be contacted in case of need; developing perspective-taking skills; recognition of the group as a resource. Exercises: “Circles of connection” ^1^, “Social DNA-V”, “Our group in a ball of yarn”, “My hand”.
Session 8. Follow-up.	Reflecting on how participants feel and what has changed in their lives; reviewing what has been experienced and reinforcing skills learned through the intervention; collecting feedback about the intervention. Exercises: “Booster exercise” ^1^, “The island of the self”.

^1^ Exercise taken from Hayes and Ciarrochi’s DNA-V manual [49] after permission. ^2^ Blended approach only. ^3^ Not present in the Blended approach. ^4^ Home activity in the Blended approach.
