# Supplementary material for: Promoting Mental Health and Well-Being among Adolescent Young Carers in Europe: A Randomized Controlled Trial Protocol
Source: Int J Environ Res Public Health. 2021 Feb 19;18(4):2045. doi: 10.3390/ijerph18042045 (PMC7922705; doi:10.3390/ijerph18042045)
Supplement: Supplementary file 1 [file ijerph-18-02045-s001.pdf]

Table S1. Session-by-session outline of the ME-WE intervention program for COVID-19-related online delivery.

Session	Content
Session 1. Getting to know each other and intervention presentation.	Welcome, introduction of facilitators and participants; establishing program engagement and group rules. Introduction to DNA-V model and overview of the intervention; collecting participants' expectations. Exercises: "DNA-V model description"[1], "Dropping the anchor".
Session 2. The Advisor: dealing with annoying thoughts.	Introduction to the concept of annoying thoughts and the futility of trying to control them; learning to have a different, healthier relationship with these thoughts: letting them go in and out without being trapped in them; learning not to identify oneself with Advisor's statements; introduction to the concept of mindfulness and first practice. Introduction to the online environment of the ME-WE app.[2] Exercises: "Give the Advisor a microphone"[1], "Normalize the Advisor – The GPS metaphor"[1], "Going to extremes"[1], "Balloon breathing"[3]. Home activity: "Watching for the Advisor"[1].
Session 3. The Noticer: being in connection with our feelings.	Identifying and getting in touch with our emotions, the body and the physical signals that come from the world around us, and understanding the importance of connecting with the present moment; developing understanding about the futility of trying to control unwanted feelings; learning to acknowledge feelings and allow them to be there; introduction to and practice on the AND acronym (Aware, Name, Describe). Exercises: "Rediscovering the wisdom of our feelings", "Feeling with the body - Practicing AND"[1], "Beachball metaphor"[1], "Body-scan mindfulness exercise".
Session 4. The Discoverer: growing and thriving.	Identifying challenging situations, encouraging the enlargement of the behavioural repertoire to make life bigger, richer, and more vital, or to refocus energy on better listening to what is important and taking committed action towards values; identifying personal strengths. Exercises: "Tracking workability of old behaviors"[1], "Strength spotting card sort"[1], "One moment meditation"[3]. Home activity: "Using the Discoverer to discover values"[1].
Session 5. Values: connecting to meaning and vitality.	Introduction to values, and recognition of the important role they play on one's life; learning to be free to listen to values and decide on actions; becoming aware of what is important in life, identifying values and goals, and committing to take action to live in line with them by developing an action plan. Exercises: "What values are"[3], "Values card sort"[1], "My valued journey"[1], "Mindfulness and music"[3]. Home activity: "My valued journey"[1].
Session 6. Developing a flexible self-view and self-compassion.	Developing a flexible self-view by experiencing oneself as a changing, evolving human, and weakening dysfunctional self-concepts; practising loving-kindness toward oneself and self-compassion by learning to forgive oneself and working on guilt and self-criticism.

	Exercises: "Strengthening my self-view"[1], "Becoming a friend to yourself"[1], "Compassionate letter"[3], "3 minutes breathing space"[4], "A gentle action".<br>Home activity: "A gentle action".
Session 7. Building strong social networks.	Identifying what is important in a relationship and especially in a caring one; building strong social networks by becoming aware of people who can support and can be contacted in case of need; developing perspective-taking skills; recognition of the group as a resource.<br>Exercises: "Circles of connection"[1], "Social networking using Conversation Cards"[1], "Our group in a ball of yarn", "My hand"[2].
Session 8. Follow-up.	Reflecting on how participants feel and what has changed in their lives; reviewing what has been experienced and reinforcing skills learned through the intervention; collecting feedback about the intervention.<br>Exercises: "Booster exercise"[1], "The island of the self".

[1] Exercise taken from Hayes and Ciarrochi's DNA-V manual [49] after permission.

[2] Blended approach only.

[3] Home activity in the Blended approach.

[4] Not present in the Blended approach.
